# Neural substrates of predicting anhedonia symptoms in major depressive disorder via connectome‐based modeling

**DOI:** 10.1111/cns.14871

**Published:** 2024-07-22

**Authors:** Tingyu Yang, Yangpan Ou, Huabing Li, Feng Liu, Ping Li, Guangrong Xie, Jingping Zhao, Xilong Cui, Wenbin Guo

**Affiliations:** ^1^ Department of Psychiatry, National Clinical Research Center for Mental Disorders, and National Center for Mental Disorders The Second Xiangya Hospital of Central South University Changsha China; ^2^ Department of Child Healthcare Hunan Children's Hospital Changsha China; ^3^ Department of Radiology The Second Xiangya Hospital of Central South University Changsha China; ^4^ Department of Radiology Tianjin Medical University General Hospital Tianjin China; ^5^ Department of Psychiatry Qiqihar Medical University Qiqihar China

**Keywords:** anhedonia, connectome‐based predictive modeling (CPM), major depressive disorder (MDD), melancholic and non‐melancholic depression, support vector machines (SVM)

## Abstract

**Main Problem:**

Anhedonia is a critical diagnostic symptom of major depressive disorder (MDD), being associated with poor prognosis. Understanding the neural mechanisms underlying anhedonia is of great significance for individuals with MDD, and it encourages the search for objective indicators that can reliably identify anhedonia.

**Methods:**

A predictive model used connectome‐based predictive modeling (CPM) for anhedonia symptoms was developed by utilizing pre‐treatment functional connectivity (FC) data from 59 patients with MDD. Node‐based FC analysis was employed to compare differences in FC patterns between melancholic and non‐melancholic MDD patients. The support vector machines (SVM) method was then applied for classifying these two subtypes of MDD patients.

**Results:**

CPM could successfully predict anhedonia symptoms in MDD patients (positive network: *r* = 0.4719, *p* < 0.0020, mean squared error = 23.5125, 5000 iterations). Compared to non‐melancholic MDD patients, melancholic MDD patients showed decreased FC between the left cingulate gyrus and the right parahippocampus gyrus (*p*__bonferroni_ = 0.0303). This distinct FC pattern effectively discriminated between melancholic and non‐melancholic MDD patients, achieving a sensitivity of 93.54%, specificity of 67.86%, and an overall accuracy of 81.36% using the SVM method.

**Conclusions:**

This study successfully established a network model for predicting anhedonia symptoms in MDD based on FC, as well as a classification model to differentiate between melancholic and non‐melancholic MDD patients. These findings provide guidance for clinical treatment.

## Graphical abstract

Our study aimed to identify a predictive model for anhedonia symptoms using connectome‐based predictive modeling (CPM) in major depressive disorder (MDD). The abnormal functional connectivity patterns between the default mode network and the limbic network in the predictive networks serve as potential neurobiological markers to distinguish between melancholic and non‐melancholic MDD. These findings revealed distinct neural substrates for anhedonia symptoms in melancholic and non‐melancholic MDD.

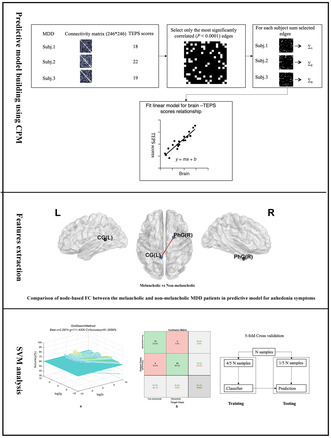

## 1 INTRODUCTION

Anhedonia, characterized by the inability to experience pleasure or loss of interest in previously enjoyable activities,^1^ is a pivotal symptom in the diagnostic criteria for major depressive disorder (MDD). Its significance is emphasized by both the International Classification of Diseases‐10^2^ and the Diagnostic and Statistical Manual of Mental Disorders‐IV Edition (DSM‐IV). Anhedonia in MDD is associated with increased disease severity, higher risk of relapse and suicide, and poor response to interventions.^1^, ^3^, ^4^ Comprehending the neural mechanisms that underlie anhedonia holds significant promise for elucidating the pathophysiology of MDD and could potentially guide the refinement of treatment approaches for MDD.

Resting‐state functional magnetic resonance imaging (fMRI) is a feasible approach to explore the neural mechanisms of anhedonia in MDD. It can noninvasively reveal the functional connectivity (FC) between different brain regions.^5^ Previous studies have found a correlation between abnormal FC patterns and anhedonia in MDD. For example, Lu et al.^6^ observed increased FC in the bilateral superior frontal gyrus in MDD patients with anhedonia compared to MDD patients without anhedonia. Yang et al.^7^ discovered a positive correlation between the severity of anhedonia and the extent of ventral caudate FC with the cuneus and middle temporal gyrus in MDD patients. Other studies have also reported heightened FC in the frontal‐limbic circuits of MDD patients with anhedonia.^8^, ^9^ However, the research outcomes have been inconsistent, and these abnormal FC are widely distributed across various brain regions.^10^, ^11^ Therefore, this study aimed to establish a predictive networks model for anhedonia symptoms using whole‐brain FC patterns. The improvement in diagnosing and predicting anhedonia in MDD patients can be achieved by identifying abnormal FC features and constructing predictive models. This facilitates early intervention and treatment. However, it is imperative to acknowledge the existing dearth of research in this particular domain.

Prior evidence supports the possibility that melancholic and non‐melancholic MDD represent two distinct biological subtypes. For instance, our previous research revealed differences in spontaneous brain activity at the location of the left anterior cingulate cortex between melancholic and non‐melancholic MDD patients,^12^ and distinct voxel‐mirrored homotopic connectivity in the precentral gyrus and precentral/postcentral gyrus was observed between these two subtypes.^13^ Regarding treatment response, the presence of melancholic symptoms has been positively associated with the effectiveness of electroconvulsive therapy, tricyclic antidepressants,^14^ and fluoxetine treatment.^15^ We aimed to identify differences in the predictive networks model for anhedonia symptoms between melancholic or non‐melancholic MDD, thereby uncovering potential neurobiological mechanisms underlying these two subtypes of MDD, which has significant implications for accurate diagnosis and personalized treatments.

Machine learning‐based classification and prediction demonstrate immense potential in the identification of neurobiological biomarkers. These biomarkers not only hold clinical significance but also have the potential to serve as prognostic indicators for MDD.^16^ Recently, various machine learning techniques have been applied to predict clinical outcomes of psychiatric disorder patients based on MRI data.^17^, ^18^ Connectome‐based predictive modeling (CPM) is a data‐driven, machine learning technique, and has been successfully used to predict treatment response in cocaine use disorder,^19^, ^20^ treatment response in depression disorder,^17^, ^21^ and severity of anxiety symptoms.^22^ This indicates that CPM is suitable for using FC to predict anhedonia symptoms in MDD patients. These FC can be referred to as connectome “fingerprints”,^23^ which can serve as important features to distinguish between melancholic and non‐melancholic MDD patients. Moreover, support vector machine (SVM) algorithm, a classification machine learning method, was widely used in MDD researches due to its ability to process high‐dimensional data and high classification accuracy.^24^, ^25^, ^26^ The advantages of choosing SVM as the classification model are as follows. First, SVM is suitable for both linear and nonlinear problems and offers a variety of built‐in kernel functions. Second, SVM performs well with small samples. Third, SVM differentiates between samples of different classes by finding the optimal hyperplane, which has a clear geometric interpretation. Finally, SVM focuses mainly on support vectors, making it less susceptible to outliers, which is crucial for small sample data.

Therefore, the present study aimed to establish a predictive model for anhedonia symptoms using CPM in MDD. Subsequently, we explore the differences in predictive networks for anhedonia symptoms between the two subtypes of MDD (melancholic and non‐melancholic MDD). Finally, the SVM method was employed to determine whether these different FC patterns could serve as potential neurobiological markers to classify melancholic and non‐melancholic MDD patients. This study will contribute to uncovering the underlying neurobiological mechanisms of anhedonia symptoms in MDD and explores the intrinsic FC patterns of its two distinct subtypes.

## 2 METHODS

### 2.1 Participants

The study recruited 64 patients diagnosed with MDD and 32 healthy controls (HCs). Five MDD patients were excluded due to excessive head movement. The patients were recruited from the Second Xiangya Hospital of Central South University. Diagnosis of MDD was based on the DSM‐IV criteria. Within the MDD patient group, there were 31 patients with melancholic MDD and 28 patients with non‐melancholic MDD. Inclusion criteria for melancholic MDD patients included the pervasive anhedonia or unreactive mood and at least three characteristic symptoms such as depression distinct from grief or loss, worsened morning mood, early morning awakening, appetite or weight disturbances, psychomotor difficulties, and excessive guilt. HCs were recruited from the community and matched with the patient group in terms of age and sex. Exclusion criteria for both the MDD and HCs groups included (1) no previous or current psychiatric disorders (except for MDD in MDD group), neurological disorders, and substance abuse; (2) excluding brain structural abnormalities, severe physical illnesses, pregnancy, and other contraindications to fMRI; (3) HCs did not have first‐degree relatives with any psychiatric disorders. All MDD patients included in the study were experiencing their first episode of MDD and were not receiving any medication prior to the neuroimaging session. Ethical approval for data collection was obtained from the Medical Research Ethics Committee of the Second Xiangya Hospital, China, and an informed consent was obtained from all participants.

### 2.2 Symptom assessment

Depression severity was evaluated by the psychiatrist‐assessed Hamilton Depression Rating Scale (HAMD, 17 items).^27^ Higher HAMD scores indicate more severe depressive symptoms. Anhedonia was evaluated by the Temporal Experience of Pleasure Scale (TEPS).^28^, ^29^ Lower TEPS scores indicate more severe anhedonia. See Data S1 for details.

### 2.3 Image acquisition and preprocessing

fMRI scanning was performed at the Second Xiangya Hospital of Central South University using a 3.0‐T Siemens MRI Scanner. During the experiments, the participants were instructed to relax with their eyes closed, without falling asleep. Resting‐state functional imaging data were obtained utilizing a gradient echo‐planar imaging (EPI) sequence (repetition time: 2500 ms, echo time: 25 ms, field of view: 240 × 240 mm, flip angle: 90°, matrix size: 64 × 64, 3.5 mm slice thickness, no gap, 39 slices, and 200 volumes). See Data S1 for details.

### 2.4 FC matrices

The fMRI data were parcellated into 246 regions of interest (ROIs) utilizing the Human Brainnetome Atlas (BNA),^30^ which delineates the brain into 210 cortical and 36 subcortical subregions based on anatomical and FC information. Subsequently, representative time series were computed for each ROI by averaging the fMRI time series across all voxels within the respective region. This resulted in the generation of a participant‐specific 246 × 246 FC matrix, representing the connectivity strengths between each pair of ROIs.

### 2.5 CPM analysis

CPM analysis was conducted using MATLAB scripts.^23^ Please refer to the Data S1 for specific step‐by‐step instructions.

### 2.6 Node‐based FC analysis

In the CPM framework, multiple edges are usually present for selection. To address this issue, we adopted a strategy that involved the identification of high‐degree nodes, or nodes with a greater number of edges.^19^ These highest‐degree nodes were then utilized as seeds, and their FC with other nodes was computed.

### 2.7 SVM analysis

In this study, the LIBSVM software^31^ was employed to evaluate the discriminative capability of abnormal FC values in brain regions. Specifically, the performance of SVM was evaluated in distinguishing melancholic MDD from non‐melancholic MDD. The SVM analysis involved these steps: data collection, splitting samples into training and test sets (using five‐fold cross‐validation), normalizing features to [−1, +1], selecting gaussian radial basis function kernels for classification. These kernels have two parameters, *c* and *g*, optimized using grid search and cross‐validation. Validation was done via five‐fold cross‐validation, where samples were randomly split into five groups. Four groups were for training and one for testing.^32^

### 2.8 Statistical analyses

The normality test for continuous data was performed using histograms and the Shapiro–Wilk test. All data conform to a normal distribution. Among the melancholic MDD, non‐melancholic MDD, and HCs groups, between‐group comparisons of continuous variables were conducted using one‐way analysis of variance, while gender distribution was examined via chi‐square test. Post‐hoc tests were performed for pairwise comparisons. An independent samples *t*‐test was utilized to investigate differences in node‐based FC between melancholic and non‐melancholic MDD patients, with Bonferroni correction applied. Partial correlation analysis was applied to analyze the correlation between the abnormal node‐based FC and clinical variables of the MDD patients with age, education level, illness duration, and framewise displacement serving as covariates, also adjusted with Bonferroni correction. The significance level was set at a two‐tailed *α* of 0.05.

## 3 RESULTS

### 3.1 Demographic and clinical variables of participants

Demographic and clinical data are presented in Table 1. Post‐hoc analysis showed that, except for TEPS contextual anticipatory score (*p* = 0.688), the MDD group exhibited significantly lower scores than the HCs across all other TEPS subscales and total scores (all *p* < 0.05). Additionally, there was no significant difference in HAMD scores between the melancholic and non‐melancholic MDD patients (*p* = 0.779). The scores for total TEPS, TEPS anticipatory, TEPS abstract anticipatory and TEPS contextual anticipatory were higher in non‐melancholic MDD patients compared to melancholic MDD patients (all *p* < 0.05).

**TABLE 1:** Demographic and clinical characteristics of MDD and HCs groups.

Variables	MDD (*n* = 59)		HCs (*n* = 32)	*F*, *t* or *χ* ^2^	*p*	Follow‐up tests
	Melancholic (*n* = 31)	Non‐melancholic (*n* = 28)				
Age (years)	28.65 **±** 5.30	32.04 ± 8.18	29.59 ± 5.00	2.291	0.107	
Gender (male/female)	10/21	10/18	15/17	1.550	0.461	
Education (years)	15.16 ± 3.195	12.54 ± 3.00	14.59 ± 2.82	6.143	0.003	
Illness duration (months)	6.75 ± 4.26	5.96 ± 4.64		0.462	0.500	
FD	0.05 ± 0.03	0.04 ± 0.02	0.05 ± 0.02	0.373	0.690	
HAMD	21.77 ± 3.79	21.00 ± 3.14	0.94 ± 0.95	527.891	<0.001	MDD > HCs
TEPS anticipatory	26.30 ± 7.83	33.71 ± 5.91	35.13 ± 6.11	24.686	<0.001	MDD < HCs, Melancholic < Non‐melancholic
Abstract anticipatory	13.17 ± 4.71	17.04 ± 3.85	19.94 ± 2.7	5.951	0.004	MDD < HCs, Melancholic < Non‐melancholic
Contextual anticipatory	13.13 ± 3.90	16.68 ± 3.64	15.19 ± 4.31	18.186	<0.001	Melancholic < Non‐melancholic
TEPS consummatory	32.00 ± 7.34	35.75 ± 7.12	42.53 ± 6.63	14.863	<0.001	MDD < HCs
Abstract consummatory	20.20 ± 5.12	22.39 ± 5.28	26.78 ± 4.23	10.947	<0.001	MDD < HCs
Contextual consummatory	11.80 ± 3.18	13.36 ± 3.27	15.75 ± 3.65	19.527	<0.001	MDD < HCs
TEPS total score	58.30 ± 13.95	69.46 ± 11.16	77.66 ± 11.6	64.191	<0.001	MDD < HCs, Melancholic < Non‐melancholic

Abbreviations: FD, framewise displacement; HAMD, 17‐item Hamilton Rating Scale for Depression; HCs, healthy controls; TEPS, temporal experience of pleasure scale.

### 3.2 Prediction of the anhedonia symptoms in MDD patients

**FIGURE 1:**
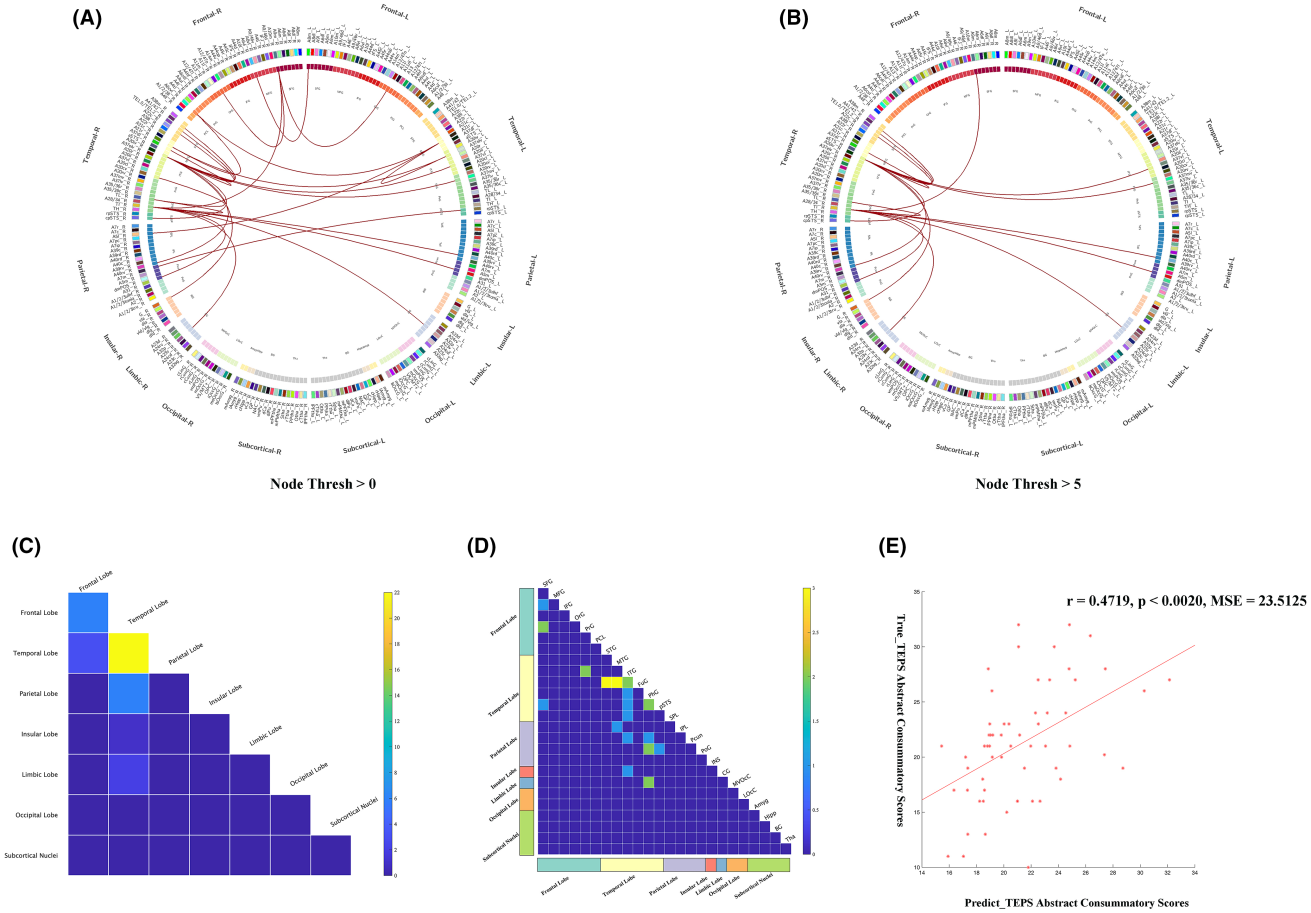
Visualizing selected connectivity features. Image (A) (Node Threshold > 0) represents 26 edges positive correlated with the TEPS Abstract Consummatory scores with a typical significance threshold of *p* = 0.0001. Image (B) shows nodes with edge connections greater than 5. (C) and (D) Matrix plots: The cells of the matrices plots represent the total number of positive edges connecting the nodes in different brain regions. The images (C) and (D) represent seven different brain lobes and 24 different brain gyrus of the Human Brainnetome Atlas (BNA) template, respectively. (E): The CPM model predicted the TEPS Abstract Consummatory scores of the MDD patients (*r* = 0.4719, *p* < 0.0020, 5000 iterations). For details on the division of brain regions, please refer to the http://atlas.brainnetome.org/bnatlas.html. CPM, connectome‐based predictive modeling; MDD, major depressive disorder; TEPS, temporal experience of pleasure scale.

CPM could successfully predict anhedonia symptoms in MDD (positive network: *r* = 0.4719, *p* < 0.0020, mean squared error (MSE) = 23.5125, 5000 iterations). The Figure 1 showed that 26 edges were positively related to the TEPS abstract consummatory score with a typical significance threshold of *p* = 0.0001. The predictive networks model primarily focuses on connections between the temporal, frontal, parietal, and occipital lobes. The nodes in right inferior temporal gyrus (ITG) and right parahippocampal gyrus (PhG_R) with five or more edges and were the highest‐degree nodes (Table 2, Table S1 and Figure 1). We designated these two nodes as seed points and calculated node‐based FC.

**TABLE 2:** Highest‐degree nodes and their connections in the positive network predictive of TEPS abstract consummatory score in MDD group.

Network	Connections							
	Node	Frontal Lobe	Temporal Lobe	Parietal Lobe	Insular Lobe	Limbic Lobe	Occipital Lobe	Subcortical Nuclei
Positive	ITG_R		STG_R (3)	IPL_ R	INS_R			
			MTG_R					
			ITG_L					
			FuG_L					
			pSTS_R					
	PhG_R	SFG_R	PhG_R	IPL_L		CG_L		
				PCun_L		CG_ R		
				PCun_R				

*Note*: The division of brain regions is based on the Human Brainnetome Atlas (BNA) template.

Abbreviations: CG, cingulate gyrus; FuG, fusiform gyrus; INS, insular gyrus; IPL, inferior parietal lobule; ITG, inferior temporal gyrus; MDD, major depressive disorder; MTG, middle temporal gyrus; Pcun, precuneus; PhG, parahippocampal gyrus; pSTS, posterior superior temporal sulcus; SFG, superior frontal gyrus; STG, superior temporal gyrus.

### 3.3 Comparison of node‐based FC between the melancholic and non‐melancholic MDD patients

**FIGURE 2:**
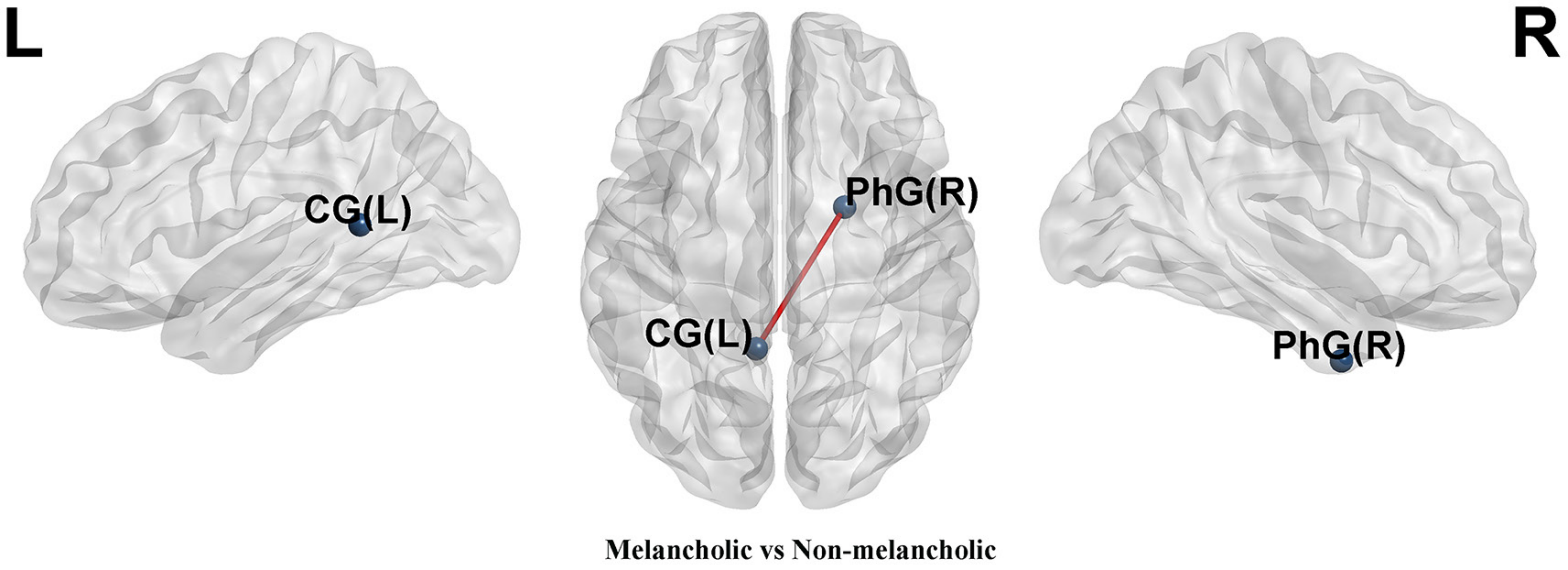
Comparison of node‐based FC between melancholic MDD patients and non‐melancholic MDD patients. The blue spheres represent nodes, the red lines represent positive FC, and the statistical results have been corrected using the Bonferroni method. CG, cingulate gyrus; PhG, parahippocampal gyrus.

Compared to non‐melancholic MDD patients, melancholic MDD patients showed decreased FC between the left cingulate gyrus (CG_L) and the PhG_R (*p*__bonferroni_ = 0.0303) (Table S2 and Figure 2).

### 3.4 SVM analyses

**FIGURE 3:**
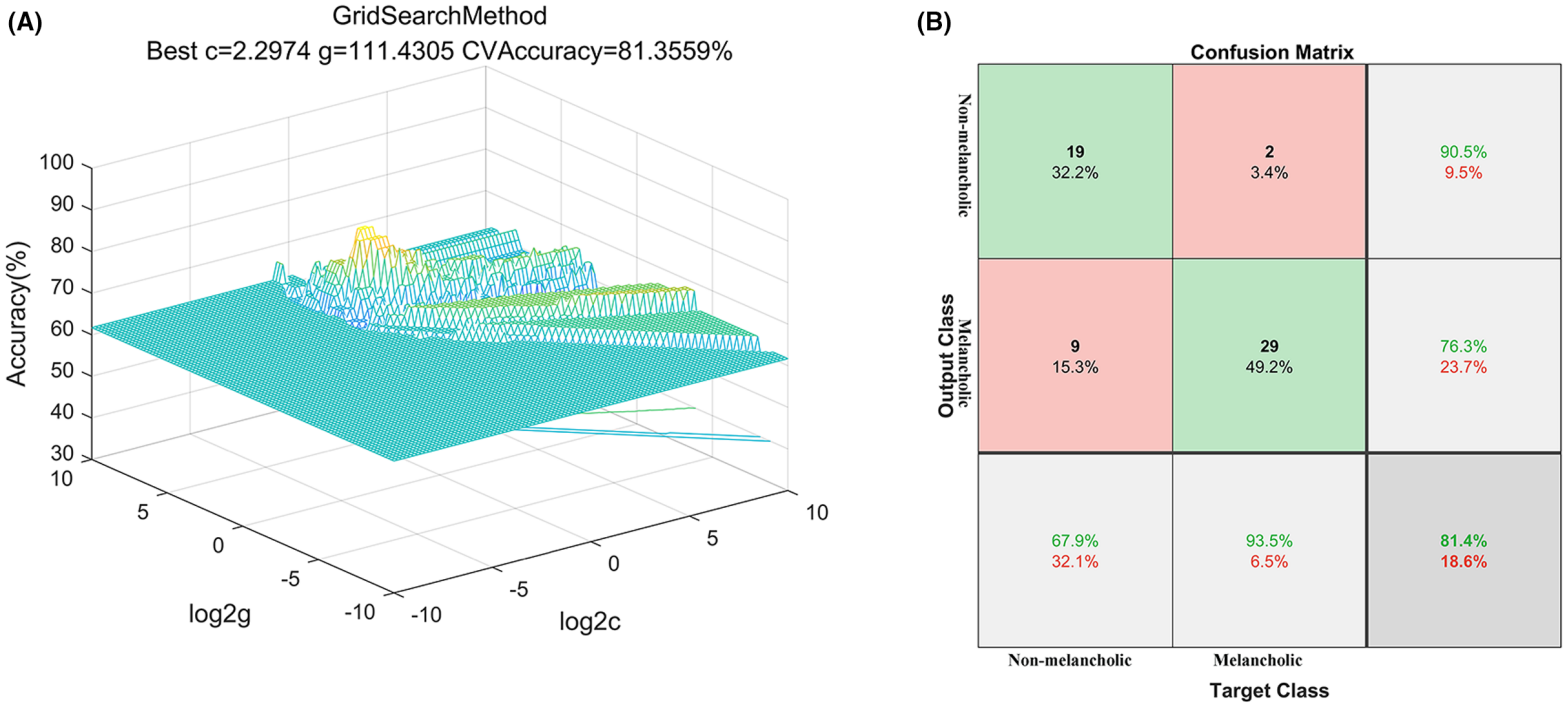
Visualization of classifications through SVM using the FC values between PhG_R and CG_L to discriminate the melancholic MDD patients from the non‐melancholic MDD patients. Left: 3D view of the classified accuracy with the best parameters using seed‐based FC. Right: Confusion Matrix. The target class represents the true classification. The output class represents the predicted classification. The sensitivity, specificity, and accuracy of SVM classification are 93.54% (29/31), 67.86% (19/28), and 81.36% (48/59) respectively. SVM, support vector machine.

The SVM analysis revealed that FC values between PhG_R and CG_L could effectively discriminate between melancholic and non‐melancholic MDD patients, yielding a sensitivity of 93.54%, a specificity of 67.86%, and an accuracy of 81.36% (Figure 3). Under optimal parameters, the standard errors (SEs) of the accuracy, sensitivity, and specificity of the SVM classification are 0.031, 0.028, and 0.067, respectively.

### 3.5 Correlations between the FC values and anhedonia symptoms

**FIGURE 4:**
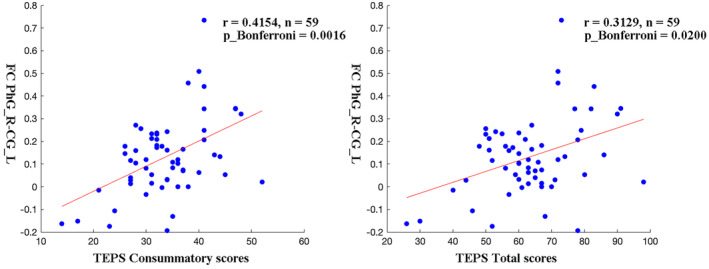
Correlations between abnormal FC values and clinical variables. Pearson correlation analysis was used to calculate the correlation. Age, education level, illness duration, and framewise displacement were used as covariates. The *p*‐values have been adjusted using Bonferroni correction. CG, cingulate gyrus; PhG, parahippocampal gyrus; TEPS, temporal experience of pleasure scale.

It was found that after controlling for age, education level, illness duration, and framewise displacement, FC values between PhG_R and CG_L were positively correlated with TEPS consummatory scores (*p*__bonferroni_ = 0.0016) and TEPS total scores (*p*__bonferroni_ = 0.0200) in MDD patients (Figure 4).

## 4 DISCUSSION

This study applied the CPM method based on FC of the whole‐brain to establish a predictive model for anhedonia symptoms in MDD. There are several meaningful findings. First, CPM could successfully predict anhedonia symptoms in MDD. The positive predictive networks primarily focus on connections between the temporal, frontal, parietal, and occipital lobes. Second, compared to non‐melancholic MDD patients, melancholic MDD patients showed decreased FC between the default mode network (DMN, CG_L) and the limbic network (LN, PhG_R) in positive predictive networks, according to Yeo 7 network division criteria.^30^ Finally, the abnormal FC patterns between the DMN and the LN in the predictive networks serve as potential neurobiological markers to distinguish between melancholic and non‐melancholic MDD patients.

The present study indicates that the FC values can be utilized to predict the anhedonia symptoms in MDD patients. Specifically, a higher FC value represents a higher TEPS score, indicating fewer anhedonia symptoms. The right ITG and the right PhG are the two highest‐degree nodes based on the prediction network of the anhedonia symptoms. The ITG is related to emotion regulation and expression processes.^33^, ^34^ The PhG is not only important for memory formation but also involved in regulating emotional processing and emotional memory.^35^ The PhG is believed to be involved in the formation of contextual associations and episodic memory during mood initiation in MDD patients. This may contribute to the tendency of patients to engage in repetitive rumination on negative thoughts rather than experiencing pleasure.^36^, ^37^ Consistent with our findings, previous studies have identified abnormal widespread FC patterns in the ITG and PhG of MDD patients with anhedonia symptoms.^38^ Previous research also found that MDD patients exhibits abnormality in brain structure in ITG and the PhG.^39^ Lower gray matter volume and cortical thickness were showed in the right ITG among MDD patients compared with HCs,^39^, ^40^ and were associated with more severe anhedonia severity.^41^ Gray matter loss in the PhG is the most frequently observed morphometric abnormality in patients with MDD.^42^ Furthermore, structural MRI research have found that a higher risk of anhedonia is associated with thinner PhG.^43^ The diminution of cortical thickness in the ITG and PhG could potentially trigger compensatory elevations in FC among these regions and other cerebral areas, effectively mitigating the functional deficits stemming from structural alterations. It is noteworthy that the MDD subjects recruited for this study were experiencing their initial episode and had not received any pharmacological intervention, thereby minimizing confounding factors such as recurrent episodes and medication effects. These augmented FC patterns may be interpreted as etiological factors rather than mere outcomes of anhedonia symptoms in patients with MDD.

In addition, the correlation between FC and anhedonia symptoms may be related to changes in plasma C‐reactive protein levels. Research has shown that FC associated with plasma C‐reactive protein levels, including right ITG and PhG, can predict anhedonia symptoms in patients with MDD.^44^ Abstract consummatory pleasure refers to the emotional experience of something that is inherently more abstract or less concrete in nature (for example, “I enjoy taking a deep breath of fresh air when I walk outside.”). Connecting such spatial information (like “outside”) with emotional information is closely associated with the function of the PhG.^35^, ^45^ Hence, the reduced FC between the PhG and brain regions associated with anhedonia(such as the superior frontal gyrus^46^ and precuneus^47^) in MDD patients could result in compromised information processing, leading to disruptions in the perception and experience of pleasure. Of note, the FC of right ITG and right PhG also related to gene expression.^48^ Further investigation is necessary to elucidate the intricate mechanisms underlying relationships between the FC of right ITG and right PhG and anhedonia in MDD patients.

Compared with non‐melancholic MDD patients, melancholic MDD patients showed decreased FC between the PhG_R and CG_L in positive predictive networks of anhedonia symptoms. The CG, the region of the DMN, plays a crucial role in the regulation of emotions and memory, receiving inputs from the orbitofrontal cortex and amygdala, and establishing connections with the PhG.^49^, ^50^, ^51^ Our previous research has shown that higher FC corresponds to milder anhedonia symptoms. Compared to MDD patients, the increased FC between PhG_R and CG_L in HCs group also confirms this.^52^ The present study found that the FC between PhG_R and CG_L is associated with anhedonia and effectively distinguishes between melancholic MDD and non‐melancholic MDD. This might suggest that the abnormal FC between PhG_R and CG_L could be an intrinsic neural substrate for the difference in the anhedonia symptoms between melancholic MDD and non‐melancholic MDD patients. Furthermore, previous research suggested that aberrant FC in the nucleus accumbens is a critical node in melancholic MDD‐related anhedonia.^53^, ^54^ The anterior cingulate cortex (a constituent of the CG) forms a neural circuit with the nucleus accumbens. Within this circuit, neurons encode signals responsive to reward acquisition and integrate effort expenditure signals with reward anticipation to influence subsequent behavior.^55^ Nevertheless, prolonged exposure to stress disrupts this neural loop, resulting in motivational anhedonia.^55^ Integrating these findings with our study outcomes, it indicates a potential involvement of the left CG as another critical node implicated in the anhedonia present in melancholic MDD patients.

CG is recognized as a crucial node of the DMN,^50^, ^56^ whereas PhG constitutes a component of the limbic system.^57^ CG and PhG have been proposed as potential interfaces connecting the systems responsible for semantic retrieval, episodic encoding, and emotional processing.^58^ This may indicate that the FC between the CG and PhG represents the integration process of the DMN and the LN. This integration determines whether patients clinically present with melancholic or non‐melancholic MDD. Besides, it is worth noting that previous literature has found a close association between the melancholic MDD and the depression severity.^59^, ^60^ However, our current investigation did not reveal any statistically significant differences in depression severity between the two groups of MDD patients. This suggests that the FC between CG and PhG may serve as a specific biological marker for melancholic MDD, independent of the severity of depressive symptoms. Notably, MDD is considered a heterogeneous disease,^61^, ^62^ and previous research^63^ combined with the present study suggests that MDD patients with melancholic traits may represent a subtype of MDD characterized by unique neuroimaging characteristics. Additionally, this study revealed no significant inter‐group differences in TEPS abstract consummatory scores between melancholic and non‐melancholic MDD patients, indicating that changes in related FC might occur prior to clinically evident abstract consummatory anhedonia in MDD patients. This further confirms that the FC between PhG_R and CG_L is an intrinsic neural substrate for the anhedonia symptoms among different subtypes of MDD.

This study has some highlights. First, CPM was used to establish a predictive model for anhedonia symptoms in MDD. Second, two machine learning methods, CPM and SVM, were respectively employed in our study for prediction and classification purposes. Next, both melancholic and non‐melancholic MDD exhibit anhedonia symptoms, but we discovered distinct intrinsic neural substrates for anhedonia symptoms in these two subtypes of MDD, revealing differences in their FC patterns. Finally, abnormal FC patterns might serve as neural biomarkers to differentiate between these two subtypes of MDD.

However, there are some limitations to this study. First, the sample size in each group is relatively small, which may limit the generalizability of our findings. Second, the predictive model we have constructed is deficient in independent sample validation, which also limits its generalizability. Finally, the cross‐sectional nature of this study limits its ability to establish a direct relationship between FC changes and anhedonia in MDD patients. Future longitudinal studies are needed to further explore the stability of FC in predicting anhedonia and classifying melancholic and non‐melancholic MDD.

## 5 CONCLUSION

This study successfully established a positive predictive networks model for anhedonia symptoms in MDD patients. The different FC patterns between the DMN and the LN in the predictive networks serve as potential neurobiological markers to distinguish between melancholic and non‐melancholic MDD patients. These research findings reveal distinct neural substrates for anhedonia symptoms in melancholic and non‐melancholic MDD patients, offering guidance for clinical treatment approaches.

## CONFLICT OF INTEREST STATEMENT

There are no financial conflicts of interest to disclose.

## Supporting information


Data S1:

## Data Availability

The data that support the findings of this study are available from the corresponding author upon reasonable request.
