# Supplementary material for: Neural substrates of predicting anhedonia symptoms in major depressive disorder via connectome‐based modeling
Source: CNS Neurosci Ther. 2024 Jul 22;30(7):e14871. doi: 10.1111/cns.14871 (PMC11261463; doi:10.1111/cns.14871)
Supplement: Supplementary file 1 — Data S1: [file CNS-30-e14871-s001.doc]

**Supplementary materials**

**2 Method**

**2.2 Symptom assessment**

The HAMD measured depressive severity, while the Temporal Experience of Pleasure Scale (TEPS)[24, 25] assessed the degree of anhedonia. The TEPS is used to measure an individual's ability to experience and anticipate pleasure across different time frames. The TEPS consists of two subscales: ‘anticipatory pleasure’ (which measures an individual's ability to anticipate and derive pleasure from future events or activities) and ‘consummatory pleasure’ (which assesses an individual's ability to experience pleasure and positive emotions in response to actual, present experiences or rewards).

**2.3 Image Acquisition and Preprocessing**

Data preprocessing was conducted using DPABI software[29]. The first 10 volumes were removed before slice timing and head motion correction were applied. The corrected data were normalized to the MNI space (3 × 3 × 3 mm³ voxel size) and smoothed with an 8-mm full-width at half-maximum Gaussian kernel. Images were then band-pass filtered between 0.01 - 0.08 Hz and linearly detrended. We conducted regression to remove the effects of Friston-24 head motion parameters, signals from the ventricular region of interest, and the white matter-centered region. Participants with excessive head motion (more than 2 mm translation and/or 2° rotation) were excluded.

**2.5 CPM analysis**

Step 1: the data were split into the training and testing sets for cross-validation (including the data of 59 MDD patients). In our study, a leave-one-subject-out cross-validation method was employed. Step 2: correlations between edges and the clinical data (TEPS abstract consummatory scores) were computed using Pearson correlation analysis in the training set. The most relevant edges were selected for further analysis based on the significance of the linear association with the TEPS abstract consummatory scores. A series of thresholds ranging from *p* < 0.0001 to *p* < 0.01 were set to select the edges. Step 3: the selected edges were summarized for every participant in the training set to obtain a single value per subject for the positive and negative edge sets, respectively. Step 4: the model was fitted by assuming the existence of a linear relationship between the TEPS abstract consummatory scores and the single-subject summary value. The summary value was inputted into the model to obtain the predicted TEPS abstract consummatory scores for the subjects in the testing set. Predictions were made separately for each testing subject in both the positive and negative edge sets. Step 5: the accuracy of the predictive model was evaluated by comparing the predicted TEPS abstract consummatory scores for all subjects in the testing set with the true observed values. Permutation testing was conducted to assess the statistical significance of the correlation between the predicted and observed values, which provided the *p*-value for the permutation test. This study conducted 5000 permutations tests.

**SUPPLEMENTARY TABLES**

Table S1: Highest degree nodes and their connections in the positive network predictive of anhedonia symptoms in MDD patients.

| **Node Threshold > 5** | | | |
| --- | --- | --- | --- |
| **Node** | | **Connections** | |
| Label ID | Region | Label ID | Region |
| 90 | ITG_R_7_1 | 72 | STG_R_6_2 |
|  |  | 74 | STG_ R_6_3 |
|  |  | 76 | STG_ R_6_4 |
|  |  | 86 | MTG_ R_4_3 |
|  |  | 97 | ITG_L_7_5 |
|  |  | 105 | FuG_L_3_2 |
|  |  | 124 | pSTS_ R_2_2 |
|  |  | 146 | IPL_R_6_6 |
|  |  | 164 | INS_R_6_1 |
| 118 | PhG_R_6_5 | 114 | PhG_R_6_3 |
|  |  | 143 | IPL_L_6_5 |
|  |  | 151 | PCun_L_4_3 |
|  |  | 154 | PCun_R_4_4 |
|  |  | 181 | CG_L_7_4 |
|  |  | 182 | CG_R_7_4 |
|  |  | 14 | SFG_ R_7_7 |

MDD = Major Depressive Disorder, ITG = Inferior Temporal Gyrus, PhG = Parahippocampal Gyrus, SFG = Superior Frontal Gyrus, STG = Superior Temporal Gyrus, MTG = Middle Temporal Gyrus, FuG = Fusiform Gyrus, pSTS = posterior Superior Temporal Sulcus, IPL = Inferior Parietal Lobule, Pcun = Precuneus, INS = Insular Gyrus, CG = Cingulate Gyrus. The division of brain regions is based on the Human Brainnetome Atlas (BNA) template.

Table S2: Comparison of node-based functional connectivity (FC) between melancholic MDD patients and Non-melancholic MDD patients.

| Node | Connections | *P*_Bonferroni | Mean |
| --- | --- | --- | --- |
| PhG_R | CG_L | 0.0303 | Melancholic < Non-melancholic |

PhG = Parahippocampal Gyrus, CG = Cingulate Gyrus, The division of brain regions is based on the Human Brainnetome Atlas (BNA) template, For details on the division of brain regions, please refer to the http://atlas.brainnetome.org/bnatlas.html.
